# Reconstitution of human microglia and resident T cells in the brain of humanized DRAGA mice

**DOI:** 10.3389/fcimb.2024.1367566

**Published:** 2024-06-25

**Authors:** Sounak Ghosh Roy, Ahmad F. Karim, Teodor-D. Brumeanu, Sofia A. Casares

**Affiliations:** ^1^ Agile Vaccines & Therapeutics, Defense Infectious Diseases Directorate, Naval Medical Research Command, Silver Spring, MD, United States; ^2^ Henry M. Jackson Foundation for the Advancement of Military Medicine, Bethesda, MD, United States; ^3^ Department of Medicine, Division of Immunology, F. Edward Hébert School of Medicine, Uniformed Services University of the Health Sciences, Bethesda, MD, United States

**Keywords:** human microglia, human immune system, umbilical cord blood, hematopoietic stem cells, humanized DRAGA mice, human resident T cells, brain, central nervous system

## Abstract

Humanized mouse models are valuable tools for investigating the human immune system in response to infection and injury. We have previously described the human immune system (HIS)-DRAGA mice (HLA-A2.HLA-DR4.Rag1KO.IL-2RgKO.NOD) generated by infusion of Human Leukocyte Antigen (HLA)-matched, human hematopoietic stem cells from umbilical cord blood. By reconstituting human cells, the HIS-DRAGA mouse model has been utilized as a “surrogate *in vivo* human model” for infectious diseases such as Human Immunodeficiency Virus (HIV), Influenza, Coronavirus Disease 2019 (COVID-19), scrub typhus, and malaria. This humanized mouse model bypasses ethical concerns about the use of fetal tissues for the humanization of laboratory animals. Here in, we demonstrate the presence of human microglia and T cells in the brain of HIS-DRAGA mice. Microglia are brain-resident macrophages that play pivotal roles against pathogens and cerebral damage, whereas the brain-resident T cells provide surveillance and defense against infections. Our findings suggest that the HIS-DRAGA mouse model offers unique advantages for studying the functions of human microglia and T cells in the brain during infections, degenerative disorders, tumors, and trauma, as well as for testing therapeutics in these pathological conditions.

## Introduction

Microglia are resident macrophages in the brain and spinal cord that play a crucial role in maintaining the functions of the central nervous system (CNS). Microglia are constantly surveying the neural environment and exert phagocytic activities to safeguard against cellular debris, dead neurons and pathogens by rapid migration to the sites of injury or inflammation (Lenz and Nelson, 2018). Microglia are also gaining increasing recognition for involvement in various neurological diseases such as Alzheimer’s disease and Human Immunodeficiency Virus (HIV)-associated dementia (HAD) (Thompson and Tsirka, 2017; Hemonnot et al., 2019). There is evidence that microglia serve as a host reservoir for latent HIV, thereby hindering the efficiency of antiretroviral therapy and facilitating future relapse and occurrence of HIV-1 associated neurocognitive diseases (HAND) (Wallet et al., 2019). Also, chronic activation of microglia can lead to neuroinflammation implicated in the pathogenesis of several neurological disorders such as Alzheimer’s disease (AD), Parkinson’s disease (PD), Frontotemporal Dementia (FTD), Multiple Sclerosis, Aging, and Stroke. Under these conditions, microglia release pro-inflammatory cytokines and reactive oxygen species that can damage neurons and exacerbate disease progression (Thompson and Tsirka, 2017; Li and Barres, 2018; Hemonnot et al., 2019; Srinivasan et al., 2020; Xu et al., 2021). The presence of T cells in healthy brain has been a topic of controversy. Although the brain was once considered an “immune-privileged” organ impermeable to lymphocyte infiltration via the Blood-Brain barrier (BBB), major parts of the CNS including the parenchyma and the meninges are now reported to be populated by resident T cells that exhibit critical housekeeping functions such as immunosurveillance and immune responses against infections (Smolders et al., 2018; Liston et al., 2022).

The DRAGA mouse strain (HLA-A2.HLA-DR4.Rag1KO.IL2RgcKO.NOD) lacks murine adaptive and innate immunity due to the Rag1 and IL2Rgc knockout mutations respectively, which are needed for engraftment of human hematopoietic stem cells (hHSCs) in the mouse bone marrow. DRAGA mice also express transgenically human leukocyte antigens (HLA) class I (A2.1) and class II (DR*0401) molecules needed for engraftment of human pro-T cells in the mouse thymus, thymic positive selection and export of functional human T cells to peripheral lymphoid organs (Danner et al., 2011; Majji et al., 2016). DRAGA mice infused with hHSCs from umbilical cord blood (thereafter termed as HIS-DRAGA) reconstitute functional human T and B cells and respond to vaccination by eliciting specific human cellular and antibody responses (Danner et al., 2011; Majji et al., 2016, 2018; Mendoza et al., 2018; Ollerton et al., 2022). The HIS-DRAGA mice also reconstitute human epithelial and endothelial cells in the lung and other organs (Mendoza et al., 2020; Brumeanu et al., 2022) as well as human Kupffer cells and hepatocytes (Wijayalath et al., 2014), which allows to sustain infection with human pathogens including *P. falciparum* (Wijayalath et al., 2014; Majji et al., 2018), HIV (Allam et al., 2015; Kim et al., 2017; Allam et al., 2018; Ollerton et al., 2022), *Orienta tsusugamushi* (scrub typhus) (Jiang et al., 2018), influenza A (Mendoza et al., 2018, 2020), SARS-CoV-2 (Brumeanu et al., 2022), and Zika virus (Yi et al., 2017). Herein, we have examined human cell reconstitution in the brain of HIS-DRAGA mice. Our results indicate the presence of human microglia and resident T cells in the brain of HIS-DRAGA mice.

## Materials and methods

### Humanized DRAGA mice

DRAGA mice expressing transgenically HLA-A2.1 and HLA-DR0401 molecules on a Rag1KO.IL2RγcKO.NOD (NRG) background have been previously described (Wijayalath et al., 2014; Allam et al., 2015; Majji et al., 2016; Kim et al., 2017; Allam et al., 2018; Jiang et al., 2018; Majji et al., 2018; Mendoza et al., 2018, 2020; Brumeanu et al., 2022; Ollerton et al., 2022). Mice were bred at the Veterinary Service Program at WRAIR/NMRC. Housing conditions were temperature-controlled (22°C ± 1°C) on a light/dark cycle of 14 h/10 h under positive air flow pathogen-free conditions and HEPA filter change stations. Mice had *ad libitum* access to irradiated food (Purina 5053 diet) and acidified water. All bedding and caging components were autoclaved. De-identified umbilical cord bloods positive for HLA-A2.1 and HLA-DR0401 were commercially procured through the New York Blood Center (Long Island City, NY, USA (https://nybloodcenter.org/products-and-services/blood-products/research-products/). Mice were irradiated (350 rads) and injected intravenously with CD3^+^ T cell-depleted cord blood cells (EasySep Human CD3 Positive Selection Kit, cat#18051, Stem Cell Technologies) containing approximately 10^5^ human CD34^+^ hematopoietic stem cells (HSC) as determined by FACS using a mouse anti-human CD34 antibody (BD Biosciences, cat#550761). CD3 depletion of human T cells was required to avoid lethal (acute) graft-versus-host-reaction. The procedures for assessing percentages of human T and B cells by FACS on the mononuclear FSC/SSC gate using human CD3 and human CD19 Abs (BD Biosciences, cat#555339, #555413) have been previously described (16). As documented in our previous studies, >95% of hHSC-infused DRAGA mice reconstitute a human immune system by 3 to 4 months hHSC infusion. The human immune cell reconstitution in peripheral blood of HIS-DRAGA mice used in this study is shown in **Table 1**. The HIS-DRAGA mice were examined for human brain-resident cell reconstitution and function at 18–42 weeks post-infusion of the human HSCs.

**Table 1:** Human immune parameters of HIS-DRAGA mice at the time of the study.

			Peripheral Blood cell %				Cord Blood			
DRAGA	Gender	Time post-hHSCs Infusion (weeks)	hCD3^+^	hCD3^+^ hCD4^+^	hCD3^+^ hCD8^+^	hCD19^+^	^HLA	Gender	Blood Group	Rh
#1	♂	37	6.4	4.9	1.5	1.3	A	F	A	Pos
#2	♂	36	21.3	19.0	2.3	0.5	B	M	O	Pos
#3	♂	25	32.9	29.5	3.4	1.4	C	M	A	Pos
#4	♂	25	36.5	31.3	5.2	0.3				
#5	♂	25	37.3	33.2	4.1	2.0				
#6	♀	42	6.1	5.5	0.6	6.8	D	F	A	Pos
#7	♂	23	21.3	17.5	3.8	14.0	E	F	O	Pos
#8	♀	18	26.3	20.4	5.9	2.3	F	F	A	Pos
#9	♂	18	49.7	35.0	14.7	0.2	G	M	O	Pos
#10	♂	25	48.6	39.4	9.2	1.9				
#11	♂	27	56.5	51.9	4.6	17.2				
#12	♂	27	30.4	27.2	3.2	2.2				
#13	♀	24	32.8	26.6	6.2	7.0	H	F	A	Pos
#14	♀	24	5.4	4.6	0.8	44.1				
#15	♂	29	13	11	2	36.9	I	M	O	Pos
#16	♂	35	12.1	9.8	2.3	46.8				
#17	♂	35	16	13.8	2.2	43.4				
#18	♀	36	16.8	12.6	4.2	63.9				
#19	♀	36	18.8	13.5	5.3	60.8				
#20	♀	36	22.8	19.9	2.9	61.3				
#21	♂	36	9.8	8.2	1.6	60.5				
#22	♀	38	7.8	6.4	1.4	69.1	J	F	A	Pos
#23	♀	38	16.4	13.6	2.8	71.6				
#24	♀	38	20.6	13.5	7.1	41.3				
#25	♀	38	20.8	13.5	7.1	62.2				
#26	♂	29	11.3	8.1	3.2	18				
#27	♂	36	21.4	17.1	4.3	37.7				
#28	♂	36	43.4	31.6	11.8	21.9				
#29	♂	36	43.2	24.3	18.9	12.3				
#30	♂	26	27.8	20.8	5.2	2.7	K	M	O	Pos
#31	♂	28	16.7	11.5	4.3	1.52				
#32	♂	28	15.7	11.3	3.6	0.2				

^A, A01:01, A02:01, B08:01, B08:01, DR03:01, DR04:01.

^B, A01:01, A02:01, B08:01, B20:02, DR03:01, DR04:01.

^C, A02:01, A33:03, B44:02, B58:01, DR03:01, DR04:01.

^D, A01:01, A02:01, B08:01, B20:02, DR03:01, DR04:01.

^E, A02:01, A11:01, B15:01, B51:07, DR04:01, DR14:04.

^F, A01:01, A02:01, B08:01, B44:02, DR03:01, DR04:01.

^G, A01:01, A02:01, B08:01, B40:01, DR03:01, DR04:01.

^H, A02:01, A32:01, B13:01, B51:01, DR04:01, DR07:01.

^I, A02:01, A11:01, B8:01, B44:02, C05:01, C07:01, DR03:01, DR4:01.

^J, A02:01, A32:01, B44:02, B44:04, DR04:01, DR11:01.

^K, A01:01, A02:01, B08:01, B55:01, DR04:01, DR16:01.

M = Male.

F = Female.

A, O = Blood groups of the cord blood donors.

### Isolation of mononuclear cells from brain

Brain mononuclear cells were isolated as previously described (Vijaya et al., 2023) with modifications. Briefly, brains were rinsed with 1X Hank’s Balanced Salt Solution (1X HBSS-Sigma H9394) and the meninges were discarded. Brains were chopped into small pieces in prewarmed (37°C) Enzyme Digestion Mix: 4.5ml 1X HBSS (Sigma#H9394) + 250 µL Fetal Bovine Serum (Sigma#F4135) + 50 µL 1M HEPES (GIBCO#15630–080) + 70 µL DNase I (stock: 1,000 U/ml) (Sigma#D4513) + 100 µL type IA Collagenase (100mg/ml stock) (Sigma#C2674) and incubated at 37°C for 15 minutes. After incubation, the cell suspension was further homogenized by pipetting and filtered through a 70µm cell strainer (Falcon#352350). The single-cell filtrate was made up to 40 ml by adding 1X HBSS, centrifuged at 2000 rpm for 10 minutes at 4°C and cell pellets were suspended in 4 ml of 37% Percoll in HBSS in 15 ml Falcon tubes. Four ml of 70% Percoll in HBSS was underlaid below the cell suspension, and 4 ml of 30% Percoll in HBSS was added on top. On top of this gradient, 2ml of 1X HBSS was added, and centrifugation was performed at 300g for 40 min at room temperature. The interphase containing brain mononuclear cells (**Supplementary Figure S1**) was collected, washed, and used for assays.

### Flow cytometry analysis

Cells were blocked with Rat Anti-Mouse CD16/CD32 (Mouse BD Fc Block™, BD#553142) on ice for 10 minutes and cell surface stained with antibodies against human CD45 (clone #2D11), CD3 (clone #HIT3a), CD4 (clone #SK3), CD8 (clone #RPA-T8), CD19 (clone #H1B19), CD14 (clone# M5E2), CD279 (clone# MIH4), HLA-DR,DP,DQ (clone #Tu39) (all from BD Biosciences), TMEM-119 (clone # A16075D, BioLegend), and anti-Mouse IgG (H+L) (Southern Biotech#1032–02 and 1032–09]. For intracellular staining of human cytokines, cells were stimulated with 500 ng/ml PMA [Sigma#P8139] and 1µM Ionomycin (Sigma#I3909) for 4 days, followed by surface staining with human CD45 and CD3 Abs and intracellularly staining with human IFN-γ Abs (clone #B27) following the manufacturer’s instructions (Invitrogen # 00–5523-00). Cells were analyzed in the mononuclear FSC/SSC as previously described (Jiang et al., 2018).

### Immunofluorescence microscopy of brain sections

Cryopreserved brain slides (5 µm) from HIS-DRAGA mice and control (non hHSC-infused) DRAGA mice were prepared by Histoserv Inc. (Germantown, MD) and used for immunofluorescent microscopy analysis. Cryopreserved human brains sections were obtained from Zyagen (San Diego, CA). Frozen slides were rehydrated with 1X PBS twice, for 5 minutes. A Fixation/Permeabilization solution was prepared by mixing 1 volume of the fix/perm concentrate (Invitrogen # 00–5123-43), and 3 volumes of fix/perm diluent (Invitrogen # 00–5223-56), added on the slides, and incubated at room temperature for 20 minutes. Slides were washed 3 times with 1X PBS and blocked with 400µl of blocking solution [2% Bovine Serum Albumin (BSA) in 1X PBS] at 37 °C for 30 minutes. After blocking, the slides were washed 3 times with 1X PBS, and incubated with labeled hCD45 (clone#2D1), hCD3 (clone#HIT3a), CD14 (clone# M5E2), hCD279 (clone# MIH4) Abs, at 37°C for 1 hour, and washed 3 times with 1X PBS. For indirect staining, slides were incubated with purified hTMEM-119 (clone # A16075D, BioLegend), washed with 1X PBS, and incubated with labeled anti-Mouse IgG(H+L) (Southern Biotech#1032–02) diluted in 2% BSA/0.01% Evans Blue/1X PBS at 37°C for 30 minutes. After incubation with secondary antibodies slides were washed 3 times with 1X PBS, let to air-dry, and mounted in Vectashield mounting medium with DAPI [Vector Labs]. The slides were analyzed in an Olympus Fluoview FV1200 Confocal microscope and Olympus BX51 Fluorescence Microscope. The stitched image of the whole brain section (**Supplementary Figure S2**) was generated by the Olympus Fluoview FV1200 Confocal microscope software.

### T cell assays

Brain cells isolated as above were cultured in 96 flat-well plates (1 × 10^5^ per well/0.2ml) and stimulated with 500 ng/ml Phorbol 12-Myristate 13-Acetate (PMA) plus 1µM ionomycin calcium salt for 3 to 4 days. Control cultures were non-stimulated. Human cytokines secreted in cell culture supernatants were measured by Luminex (#M5000031YV, BIO-RAD) which is human validated and does not cross-react with mouse cytokines (Jiang et al., 2018).

### Phagocytosis assay

Phagocytosis by human microglial cells was examined as described (Rangaraju et al., 2018; Ramesha et al., 2020; Sudwarts et al., 2022). Briefly, cells isolated from brains of HIS-DRAGA mice (0.6x10^6^/well/100µL) were cultured in RPMI plus 10% FBS in the presence of FITC-labeled Latex beads (Sigma #L4655, 2% (w/w) for 1h at 37°C and 5% CO2. Cells were stained with hCD45 and hTMEM119 Abs and analyzed by flow cytometry.

## Results

### Human hematopoietic cell repopulation in brain of HIS-DRAGA mice

**Figure 1:**
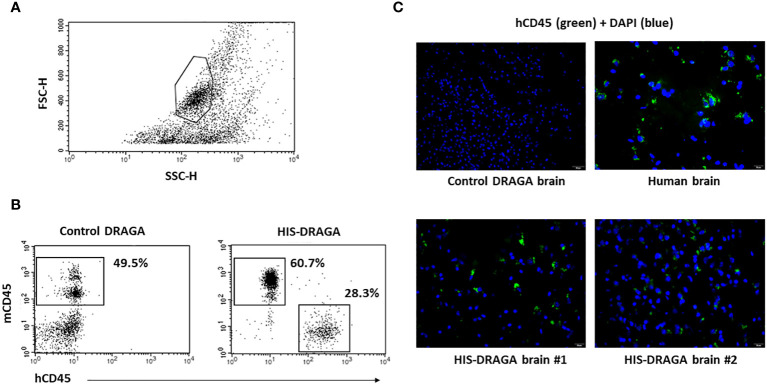
Detection and quantification of HSCs-derived hCD45+ cells in HIS-DRAGA brain. **(A)** gating of brain mononuclear cells based on the forward (FSC) and side scattering (SSC); **(B)** enumeration of human (hCD45^+^) and mouse (mCD45^+^) hematopoietic-derived cells in brains of HIS-DRAGA (right) and control DRAGA (left) mice. The brains of HIS-DRAGA, but not control, mice showed presence of hCD45^+^ cells in average 28.3% [4.4–61.1], while both groups of mice contained mCD45^+^ cells, 60.7% and 49.5%, respectively. **(C)** immunofluorescence microscopy of brain sections stained with hCD45-FITC (green fluorescence) and DAPI (cell nuclei, blue fluorescence) at 40X magnification. While the control (non-hHSC infused) DRAGA brain sections showed no specific signals (upper left), the presence of hCD45^+^ were detected in human brain (upper right) and in HIS-DRAGA mice (lower panels). The immunofluorescence images were captured at 40x magnification.

Once the reconstitution of human lymphocytes was significant in the HIS-DRAGA mice peripheral blood (mice #1–14, **Table 1**), the brain mononuclear cells were isolated as described in materials and methods section. The average number of mononuclear cells per brain was 2.2 million (interval 0.3–5.6). **Figure 1A** illustrates the gating strategy for mononuclear cells based on side (SSC) and forward (FSC) scattering. Data in **Figure 1B** (right panel) show the presence of human hematopoietic-derived hCD45^+^ cells (average 28.3%, interval 4.4–61.1) in the brain of HIS-DRAGA mice but not in control mice (left panel), while both HIS-DRAGA and control mice contained mouse hematopoietic-derived cells, mCD45^+^ (HIS-DRAGA 60.7%, interval 31.1–88.8; control DRAGA 49.5%, interval 40.1–56.2). The presence of hCD45^+^ cells in the brain of HIS-DRAGA mice and in human brain from cadavers (positive control) were further visualized by immunofluorescence microscopy. No hCD45^+^ cells were detected in brains of control (non hHSC-infused) DRAGA mice (**Figure 1C**). The results demonstrated that HIS-DRAGA mice reconstitute human cells in the brain.

#### Human microglia repopulate the brains of HIS-DRAGA mice

**Figure 2:**
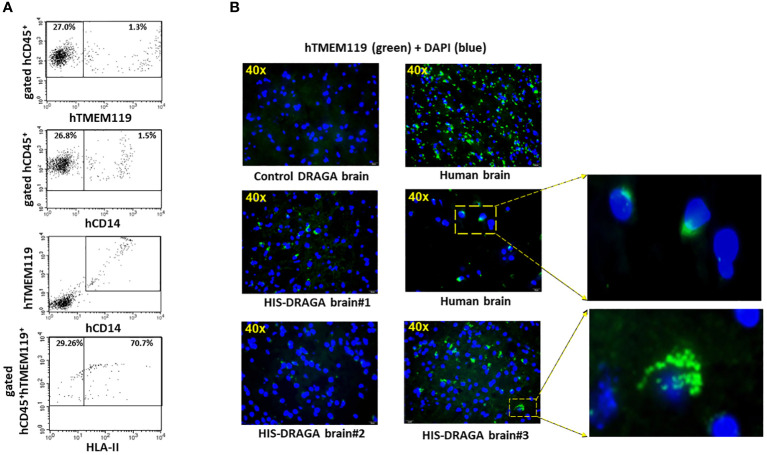
Detection and quantification of human CD45^+^TMEM119^+^CD14^+^HLA-II^+^ Microglia in HIS-DRAGA brain. Same gating strategy of cells as in **Figure 1** was used to identify and quantify human microglia in HIS-DRAGA brains (n=14). **(A)** shows the presence of hCD45^+^hTMEM119^+^ (upper panel) and hCD45^+^hCD14^+^ (middle upper panel) microglial cells in the mononuclear cell population isolated from brain of 14 HIS-DRAGA mice. Middle lower panel shows the co-expression of hTMEM-119 and hCD14 in the same cell population. Majority of human microglial cells (average 70.7%, interval 63.1–78.4) express HLA class II molecules (lower panel). **(B)** immunofluorescence microscopy of brain sections stained with hTMEM119-FITC Abs. Overlapped DAPI staining in all brain sections show the cell nuclei (blue color). While the control (non hHSC infused) DRAGA brain lacked human microglia (upper left), brain sections from humans showed uniform distribution of hTMEM119^+^ cells (green color) throughout the tissue (upper and middle right). Middle left and lower left panels show the presence of human microglial cells (hTMEM119^+^) in brains in three representative HIS-DRAGA mice. Enlargement of specific areas showing presence of human microglia in human brain and in HIS-DRAGA brain.

**Figure 3:**
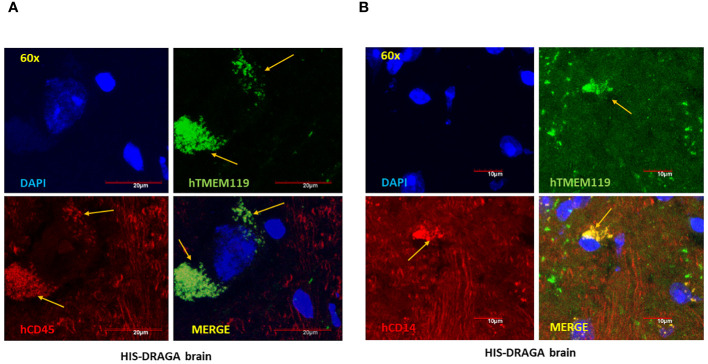
High-Resolution imaging of reconstituted human microglia in HIS-DRAGA mice brain sections. Confocal microscopy was used to obtain high magnification (60X) images of HIS-DRAGA brain sections in high-resolution. Sections were stained with hTMEM119 (green), hCD45 (red), and DAPI (blue) **(A)** or with hTMEM119 (green), hCD14 (red), and DAPI (blue) **(B)**. The data demonstrates the presence of hCD45^+^hTMEM119^+^ and hCD14^+^hTMEM119^+^ human microglial cells (yellow arrows) respectively, in HIS-DRAGA brain sections. Scale bars are shown in red.

Once established that hCD45^+^ cells were present in the HIS-DRAGA brains, we next enumerated the frequency of human microglial cells using antibodies against proteins and receptors that have been previously used as microglia-specific markers, namely, hCD45, a highly conserved receptor tyrosine phosphatase exclusively expressed on all nucleated cells of human hematopoietic origin (Rheinlander et al., 2018), hTMEM119, a type I transmembrane protein specifically expressed by resident microglia in the healthy brain (Mercurio et al., 2022), and hCD14 a toll-like receptor expressed on macrophages and monocytes (Baumann et al., 2010; Zanoni et al., 2011; Jurga et al., 2020). Data depicted in **Figure 2A** show the presence of 1.3% hCD45^+^hTMEM119^+^ (interval 0.3–3.3, upper panel ) and 1.5% hCD45^+^CD14^+^ (interval 0.3–3.3, middle upper panel) microglial cells among the mononuclear cell population from brains of HIS-DRAGA mice. **Figure 2A** middle lower panel shows co-expression of hTMEM119 and hCD14 in the gated hCD45^+^ population. The human microglial cells from HIS-DRAGA mice also expressed HLA-II molecules (average 70.7%, interval 63.1–78.3%) (**Figure 2A**, lower panel), as previously described for human microglia in the human brain (Hayes et al., 1987). The presence of human microglial cells in brains of HIS-DRAGA mice and in the human brain sections from cadavers (positive control) was further visualized by immunofluorescence using hTMEM119 antibodies, whereas no signal was detected in brains of control (non hHSC-infused) DRAGA mice (**Figure 2B**). Confocal microscopy of brain slides from HIS-DRAGA mice further demonstrated the co-expression of hCD45, hTMEM119, and hCD14 in the human microglial cells (**Figure 3**). Whole brain image showed scattered distribution of the human microglial cells (**Supplementary Figure S2**). These results indicated that HIS-DRAGA mice reconstituted human microglial cells in the brain.

#### Human microglia in HIS-DRAGA brain are phagocytic

**Figure 4:**
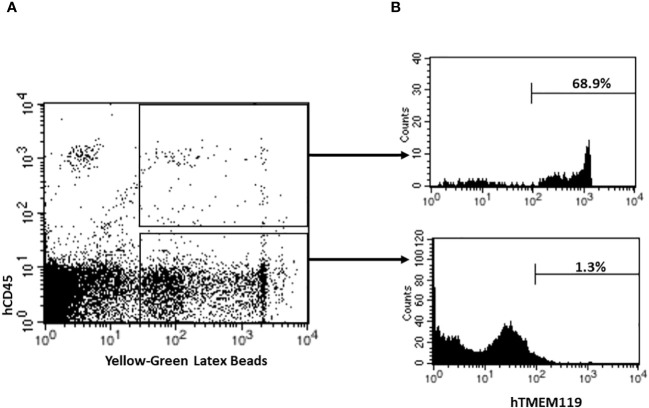
Detection and Quantification of Phagocytic Human Microglia in HIS-DRAGA mice brains. Flow cytometry was performed to analyze and quantify the percentage of phagocytic human microglial cells in HIS-DRAGA brain (n=3; mice #30–32, **Table 1**). **(A)** shows the population of human phagocytic cells (hCD45^+^Latex^+^) (upper quadrant), and mouse phagocytic cells (hCD45^-^Latex^+^) (lower quadrant). **(B)** shows expression of hTMEM119 in each of the populations described in panel A. Data shows majority of human phagocytic cells (average 68.9%, interval 46.9–86.2) are hCD45^+^hTMEM119^+^ microglial cells.

Microglia are brain specialized macrophages and the main phagocytic cells in the central nervous system (Li et al., 2022a). To determine the phagocytic function of human microglial cells from HIS-DRAGA mice, their brain mononuclear cells were cultured in the presence of fluorescent latex beads, and analyzed by flow cytometry using hCD45 and hTMEM119 Abs. As shown in **Figure 4**, majority of the human cells that internalized the latex beads (hCD45^+^Latex^+^, panel A) also expressed hTMEM119 (average 68.9%, interval 46.9–86.2, panel B) (mice #30–32, **Table 1**). Notably, the mouse microglial cells (hCD45^-^hTMEM119^-^) also showed phagocytic function. The results thus demonstrated that human microglial cells in the brain of HIS-DRAGA mice are professional phagocytes and functional as the mouse microglia.

#### Resident human T cells in brains of HIS-DRAGA mice

**Figure 5:**
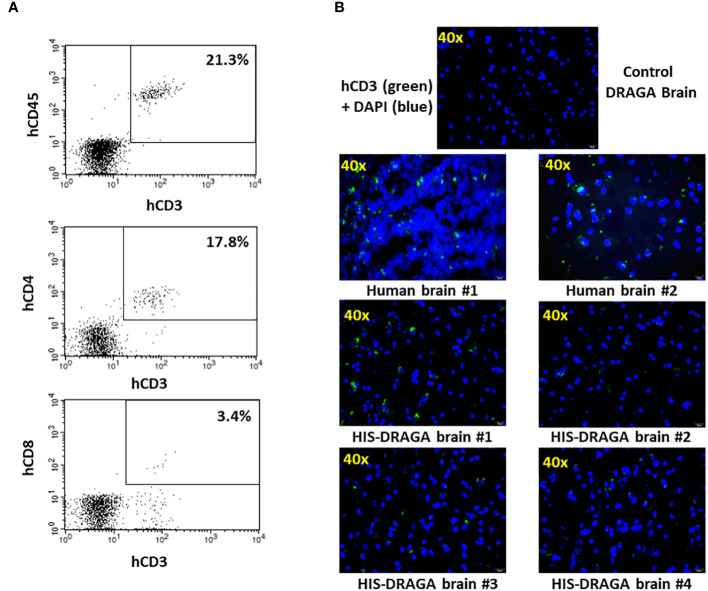
Detection and quantification of HSCs-derived human T cells in HIS-DRAGA brain. **(A)** Mononuclear cells isolated from brain of HIS-DRAGA mice (n=14) were stained with hCD45, hCD3, hCD4, and hCD8 Abs. **(A)** shows the presence of hCD45^+^hCD3^+^ T cells, hCD3^+^hCD4^+^ cells, and hCD3^+^hCD8^+^ T cells among the mononuclear cells isolated from brains; **(B)** immunofluorescence microscopy of brain sections stained with hCD3-FITC Abs (green) and DAPI (blue) shown the presence of human T cells in the brains of human cadavers and four HIS-DRAGA mice but not in control (non-hHSC infused) DRAGA mice.

**Figure 6:**
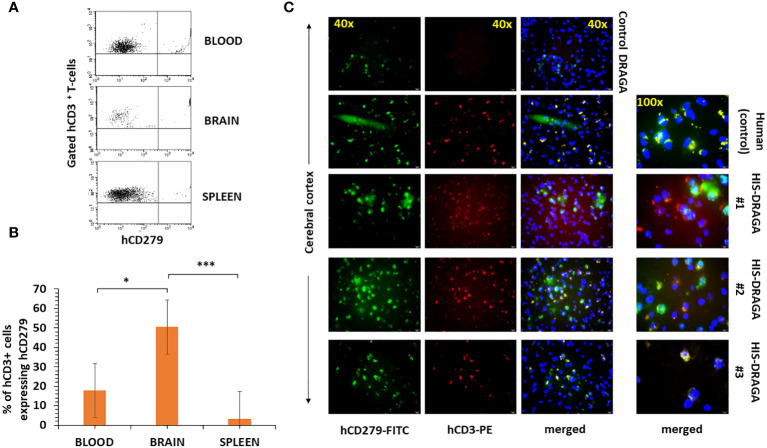
High-level expression of CD279 (PD-1) on brain-resident human T cells of HIS-DRAGA mice. Mononuclear cells from blood, brain, and spleens of HIS-DRAGA mice (n=8) were stained with hCD3 and hCD279 Abs. **(A)** shows expression of hCD279 on gated hCD3 cells in the blood (upper), brain (middle), and spleen (lower) of a representative HIS-DRAGA mouse. **(B)** frequency of hCD3^+^hCD279^+^ cells in blood (average 17.8%, interval 5.5–31.5%), brain (average 50.5%, interval 25.1–83.7%), and spleens (average 3.3%, interval 0.7–5.9%) of HIS-DRAGA mice. The frequency of hCD3^+^hCD279^+^ cells in brain of HIS-DRAGA mice was significantly higher that the counterparts human T cells in the blood (p=0.006, *) and in the spleens (p=0.0003, *** Student t-test). **(C)**, immunofluorescence microscopy of hCD3^+^hCD279^+^ double positive T cells in the human brain (positive control, second panels) and in brains of three representative HIS-DRAGA mice (three lower panels), but not in brain of control (non-hHSC infused) DRAGA mice (upper panels).

Several studies have demonstrated the presence of parenchymal T cells in the CNS of healthy human brains and disproved previous assumptions that the brain is an “immune-privileged” organ (Smolders et al., 2018; Liston et al., 2022). Since human microglial cells account only for a fraction of the total hCD45^+^ (~ 4.6%, 1.3% to 28.3%) in the brain of HIS-DRAGA mice (**Figure 2A**), we next investigated whether the remaining human cell populations may represent human resident, parenchymal T cells. Flow cytometry analyses revealed the presence of human parenchymal T cells (hCD45^+^hCD3^+^) in the HIS-DRAGA brains (average 21.3%, interval 3.6–57.6) (**Figure 5A**, upper panel). Majority of the human resident T cells belonged to the CD4 T cell compartment (average 17.8%, interval 1.8–39.0%) (middle panel), though human CD8 T cells were also detected in the brains of HIS-DRAGA mice (average 3.4%, interval 0.5–7.4%) (lower panel). No human B cells (hCD45^+^CD19^+^) were detected in the brains of HIS-DRAGA mice (data not shown). The presence of human T cells (hCD3^+^) in the brains of HIS-DRAGA mice and in the human brains from cadavers (positive control) was further visualized by immunofluorescence microscopy sing hCD3 antibodies. No signal was detected in brains of control (non-hHSC infused) DRAGA mice (**Figure 5B**). Interestingly, the human resident T cells in the brains of HIS-DRAGA mice expressed significantly higher levels of hCD279 (PD-1) as compared to their human T cell counterparts in blood (p=0.006) and in the spleen (p=0.0003) (**Figures 6A, B**), revealing a phenotypically distinct compartment of human T cells in the brain. The presence of human resident T cells co-expressing hCD3 and hCD279 was also visualized in brain sections from HIS-DRAGA mice, and in human brain from cadavers (positive control), while absent in brains from control (non-hHSC infused) DRAGA mice (**Figure 6C**). Together, the results demonstrated the presence of human T cells in the brains of HIS-DRAGA mice.

#### Human T cells in HIS-DRAGA brain secrete cytokines

**Figure 7:**
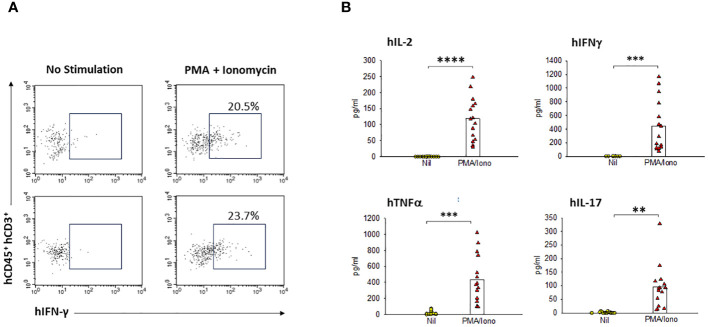
Human resident T-cells in HIS-DRAGA brains are functional and secrete human cytokines. Flow cytometry and Luminex MagPix technologies were used to quantify the cytokine profile of human T cells from HIS-DRAGA brains upon *in vitro* stimulation with PMA/Ionomycin or left unstimulated. **(A)** shows flow cytometry data demonstrating human IFNγ secretion by the stimulated T cells (right panels) as compared to unstimulated T cells (left panels) in two individual HIS-DRAGA mice. **(B)** shows significant levels of human pro-inflammatory cytokines (hIL-2, hIFNγ, hTNFα, and hIL-17) secreted in cell culture supernatants upon stimulation with PMA/Ionomycin as compared to no stimulation (Nil) as measured by Luminex. Data show cytokines levels in individual mice (n=15). No significant levels of anti-inflammatory hIL-4, h-IL10 were detected in the stimulated cultures (data not shown). ** p-value < 0.005; *** p-value < 0.0005; **** p-value < 0.00005.

Having found the presence of brain-resident human T cells, we next investigated whether these cells are functional and able to secrete human cytokines. As illustrated in **Figure 7A**, the human T cells from HIS-DRAGA mice (mice #27&28, **Table 1**) stimulated with PMA/Ionomycin secreted hIFN-γ as indicated by surface staining with hCD45, hCD3, and intracellular hIFN-γ Abs. Furthermore, the brain-resident human T cells stimulated with PMA/Ionomycin also secreted other pro-inflammatory human cytokines (IL-2, TNF-α, and IL-17) as measured by Luminex (mice#15–29, **Table 1**) (**Figure 7B**), but no significant secretion of anti-inflammatory cytokines (IL-4, IL-10) (data not shown). These results indicated that human brain-resident T cells from HIS-DRAGA mice secrete human cytokines upon activation.

## Discussion

This study presents for the first time evidence of reconstitution of human microglia and T cells in the brain of the HIS-DRAGA mouse model (HIS-DRAGA mouse: HLA-A2.HLA-DR4.Rag1KO.IL-2RγcKO.NOD). Microglia functions can be traced back in the earliest stages of mammalian brain development where they play an essential role in the development of the fetal brain and proper functioning of a healthy adult brain (Thion et al., 2018; Bohlen et al., 2019). The mechanisms by which microglia contribute to brain development involve phagocytosis of axons, dying cells, and synaptic elements in addition to other essential processes like myelination, neurogenesis, synaptic formation/maturation, and axon fasciculation (Borst et al., 2021). These events contribute to the development and refinement of neuronal circuits during brain development, regulate synaptic pruning and promote synaptic plasticity. In the healthy adult brain, microglia are instrumental in the maintenance of homeostasis primarily by regulation of synaptic communication and pruning, neuronal environment surveillance, prevention of neuronal degradation and regulation of cell-cell communication. Microglia actively interact with neurons, astrocytes, and other glial cells, and participate in complex signaling networks within the brain. These interactions are crucial for neuronal communication, synaptic connectivity, and overall homeostasis of CNS (Augusto-Oliveira et al., 2019). The dynamic profile of the microglia genome/transcriptome is a challenge for generation of an *in vitro* model, as the transfer of human microglia from the brain to a cell culture environment showed significant downregulation of microglia-specific genes (Gosselin et al., 2017). Thus, natural reconstitution of human microglial cells in the brain of HIS-DRAGA mice may allow investigations on the biology and functions of human microglia in CNS infections and degenerative disorders, tumors, and neurological trauma. Furthermore, the microglial signaling pathways relevant to the physiopathology of Alzheimer’s disease are being identified, e.g., purinergic and calcium signaling, new target therapies are being developed (Hemonnot et al., 2019; Li et al., 2022b). The HIS-DRAGA mouse reconstituting human immune system and human microglia in the brain may thus represent a new pre-clinical model for testing therapeutics. Since we previously showed that HIS-DRAGA mice sustain infection with HIV (Allam et al., 2015; Kim et al., 2017) and microglia has been considered an important HIV reservoir (Wallet et al., 2019), the HIS-DRAGA mouse model may well address brain-derived HIV pathology and its reservoirs. In addition, our results indicated that human microglial cells in brain of HIS-DRAGA mice are phagocytic, an important finding since microglial phagocytosis is crucial for both brain homeostasis as well as dysregulation during neurodegenerative CNS disorders such as Alzheimer’s Disease (AD), Parkinson’s Disease (PD), Trauma, Stroke, Amyotrophic Lateral Sclerosis (ALS) and Brain tumor formation (Galloway et al., 2019 (Janda et al., 2018; Nizami et al., 2019; Butler et al., 2021). Since microglial phagocytosis is critical in several CNS diseases (Fu et al., 2014; Miao et al., 2023) including the pathways related to microglial phagocytosis are increasingly gaining prominence) in the field of therapeutic interventions. Hence, our findings provide rationale grounds for using HIS-DRAGA as a novel pre-clinical model for potent preclinical platform for therapeutic investigations.

It was long considered that microglia and perivascular macrophages are the only resident immune cells in the brain parenchyma. However, Smolders et al. (Smolders et al., 2018) recently described the presence of brain-resident T cells in humans. We have also found human T cells among the mononuclear cells in the brain of HIS-DRAGA mice, with majority belonging to the human CD4 T cell compartment and to the lesser extend to the human CD8 T cell compartment. Since the meninges were removed before isolation of brain cells, these human T cells may well be considered brain resident cells. Furthermore, half of the human T cells in the brain of HIS-DRAGA mice expressed high levels of hCD279 (Programmed cell death protein-1, PD-1) as compared to the counterpart T cells in the blood and spleen (*p value* 0.006 and 0.0003, respectively), thus revealing a phenotypically distinct human T cell population in the brain of HIS-DRAGA mice. The human T cells in brain of HIS-DRAGA mice did not express hCD103 or hCD69 (data not shown). PD-1 is a cell surface receptor on T cells that has a role in preventing autoimmunity by promoting T cell apoptosis upon interaction with PD-L1 and PD-L2 ligands (Gong et al., 2018). PD-L1 is expressed on activated macrophages, dendritic cells, T cells and B cells, and recently found upregulated on microglia during neuroinflammation (Chauhan and Lokensgard, 2019). PD-L1 is also expressed on tumor cells and is involved in inhibiting anti-tumor activity by induction of apoptosis in tumor-specific T cells (Zheng et al., 2019). PD-1 and PD-L1 inhibitors, such as monoclonal antibodies and small molecules are currently being tested for cancer therapy in clinical trials (Ai et al., 2020). Furthermore, PD-1 also regulates the extent of neuroinflammation and the development of brain-resident memory T cells during viral (encephalitis) infection (Shwetank et al., 2019). Our study indicated that the human T cells in the brain of HIS-DRAGA mice are functional and secrete human pro-inflammatory cytokines upon stimulation, thus providing a rational ground for using these mice to investigate the role of brain-resident T cells during infections, neuroinflammation and targets for cancer PD-1 therapy as recently described (Srivastava et al., 2023). Pro-inflammatory brain cytokines play a major role in regulating neurodegenerative diseases including AD, trauma, ischemic brain injury, multiple sclerosis (MS) as well as CNS viral infections (encephalitis) (Wang et al., 2015; Ritzel et al., 2016; Shwetank et al., 2019; Konsman, 2022; Ayasoufi et al., 2023).

In conclusion, our results indicated that the HIS-DRAGA mice may serve as a novel preclinical model for investigating the role of human microglia and resident T-cells during neurodegenerative disorders, infectious diseases, and traumatic brain injury as well as for testing safety and efficacy of therapeutics in these conditions.

## Data availability statement

The original contributions presented in the study are included in the article/**Supplementary Material**. Further inquiries can be directed to the corresponding author.

## Ethics statement

The animal study was approved by Walter Reed Army Institute of Research/Naval Medical Research Command, Institutional Animal Care and Use Committee. The study was conducted in accordance with the local legislation and institutional requirements.

## Author contributions

SGR: Conceptualization, Data curation, Formal analysis, Investigation, Methodology, Validation, Writing – original draft, Writing – review & editing, Software, Visualization. AK: Writing – review & editing, Methodology, Supervision. T-DB: Conceptualization, Data curation, Formal analysis, Funding acquisition, Investigation, Methodology, Project administration, Resources, Software, Supervision, Validation, Visualization, Writing – original draft, Writing – review & editing. SC: Writing – original draft, Writing – review & editing, Conceptualization, Data curation, Formal analysis, Funding acquisition, Investigation, Methodology, Project administration, Resources, Software, Supervision, Validation, Visualization.
