# Supplementary material for: Reconstitution of human microglia and resident T cells in the brain of humanized DRAGA mice
Source: Front Cell Infect Microbiol. 2024 Jun 25;14:1367566. doi: 10.3389/fcimb.2024.1367566 (PMC11231403; doi:10.3389/fcimb.2024.1367566)
Supplement: Supplementary file 1 [file Presentation_1.pptx]

## Slide 1
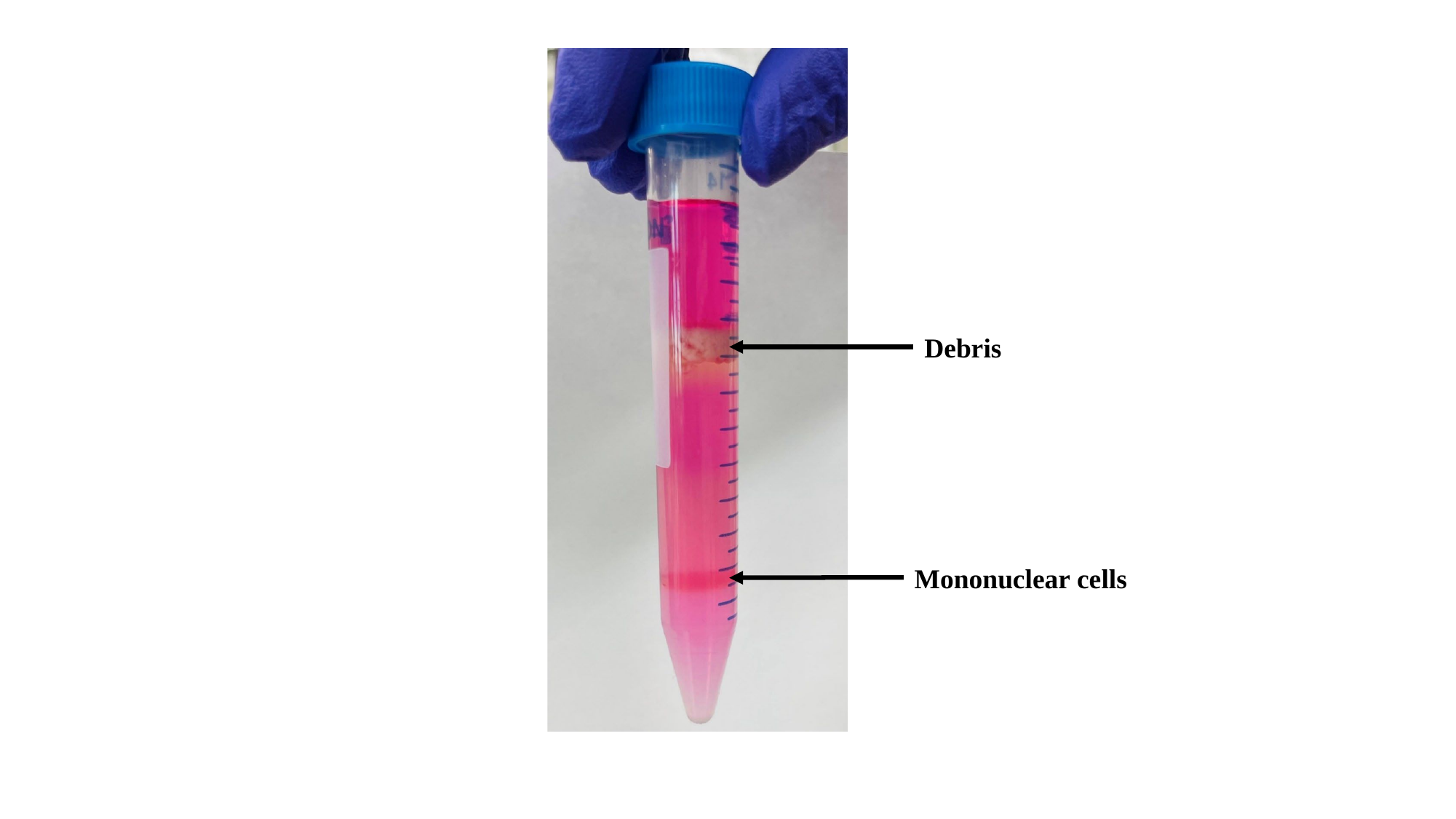

Debris
Mononuclear cells

## Slide 2
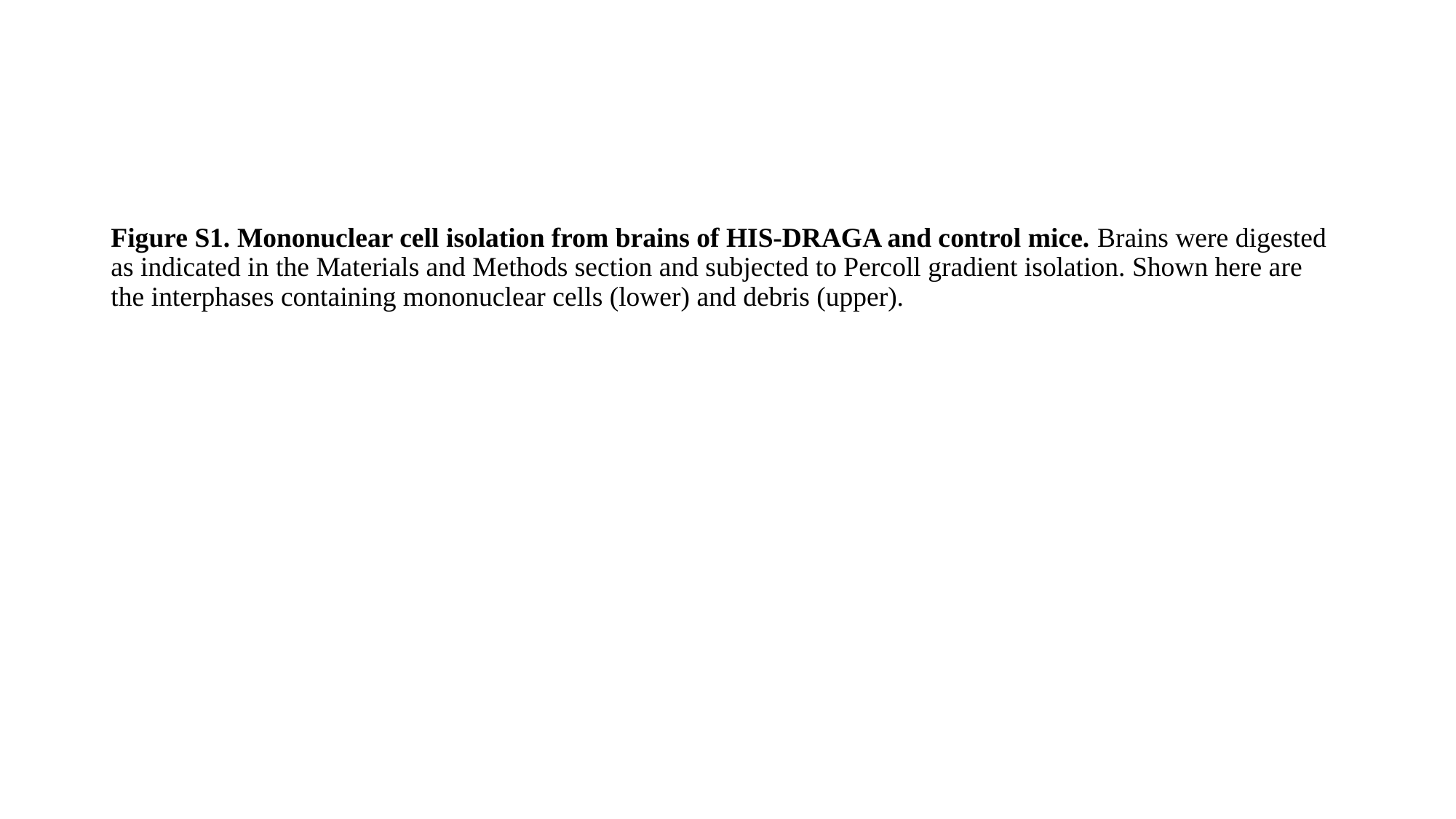

Figure S1. Mononuclear cell isolation from brains of HIS-DRAGA and control mice. Brains were digested as indicated in the Materials and Methods section and subjected to Percoll gradient isolation. Shown here are the interphases containing mononuclear cells (lower) and debris (upper).

## Slide 3
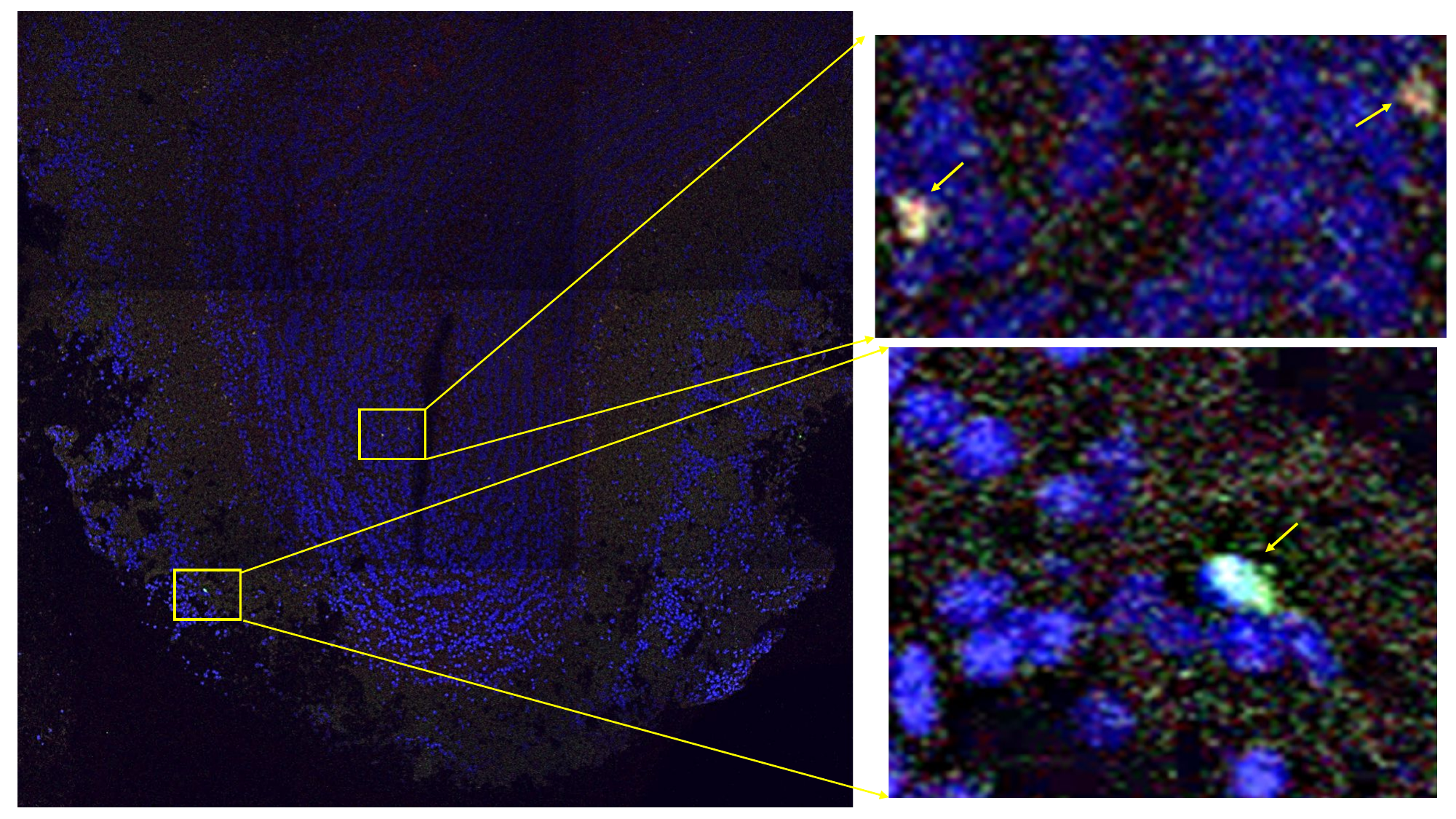

## Slide 4
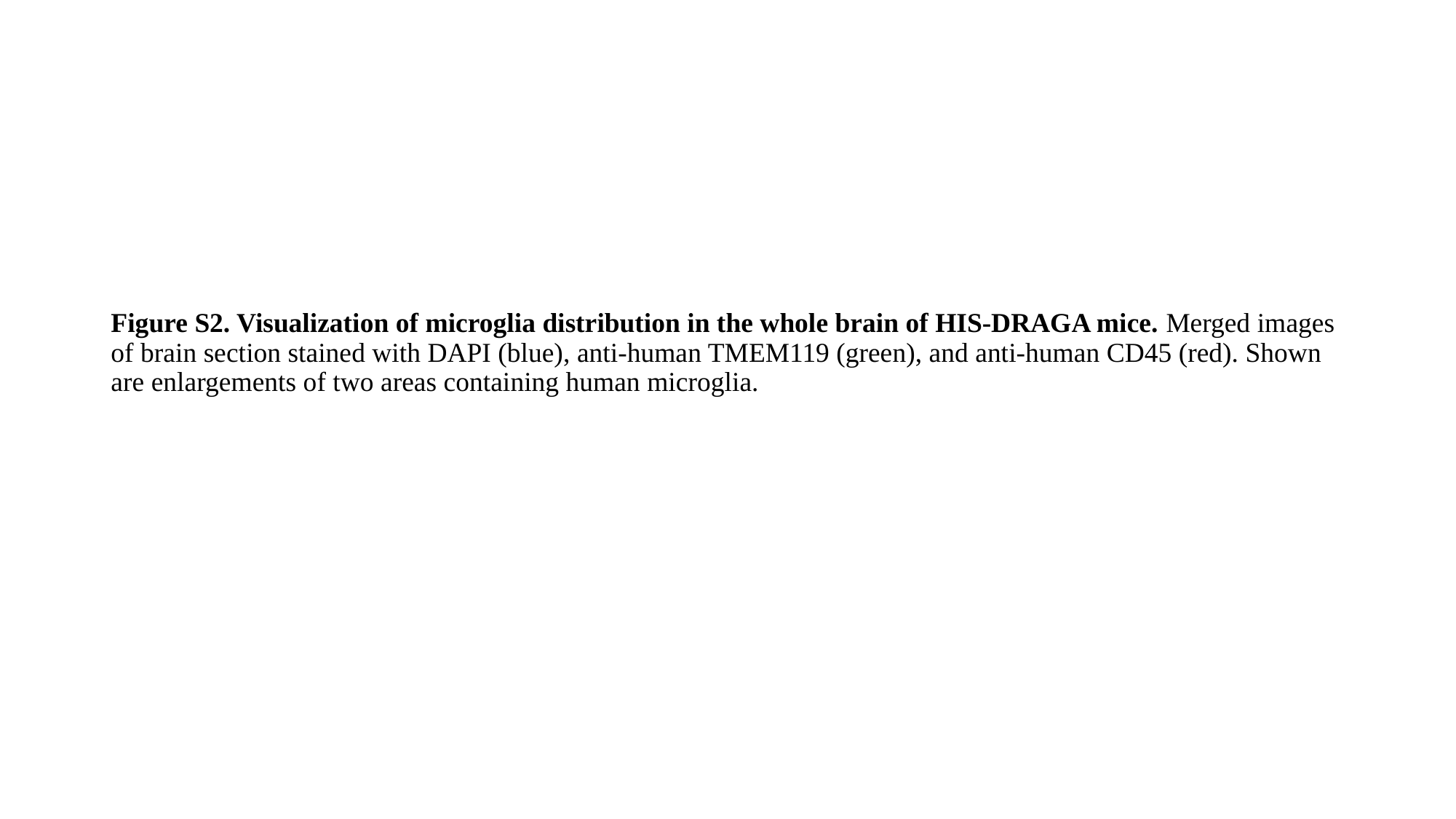

Figure S2. Visualization of microglia distribution in the whole brain of HIS-DRAGA mice. Merged images of brain section stained with DAPI (blue), anti-human TMEM119 (green), and anti-human CD45 (red). Shown are enlargements of two areas containing human microglia.
